# Postpartum Blues in Fathers: Prevalence, Associated Factors, and Impact on Father-to-Infant Bond

**DOI:** 10.3390/ijerph20105899

**Published:** 2023-05-20

**Authors:** Claire Baldy, Eloi Piffault, Margaux Chabbert Chopin, Jaqueline Wendland

**Affiliations:** 1 Laboratoire de Psychopathologie et Processus de Santé, Université Paris Cité, F-92100 Boulogne-Billancourt, France; baldy.claire@yahoo.fr (C.B.); eloi.piffault@gmail.com (E.P.); margaux.chopin@univ-tours.fr (M.C.C.); 2 Laboratory QualiPsy, University of Tours, F-37000 Tours, France

**Keywords:** postpartum blues, fatherhood, prevalence, associated factors, bonding

## Abstract

In this study we explored, in men, one of the most common postpartum syndromes in women: the postpartum blues. The aims of the study were (a) to evaluate the prevalence of postpartum blues in fathers, (b) to explore the sociodemographic and perinatal factors that may be associated with its intensity, and (c) to investigate the relationship between the intensity of blues symptoms and the quality of father-to-infant bonding. Three hundred and three French-speaking fathers living in France completed a sociodemographic and obstetrical questionnaire, the Maternity Blues Questionnaire, and the Postpartum Bonding Questionnaire. The fathers were recruited from two maternity hospitals and a Child and Maternal Health Centre within 10 days of their infant’s birth, or from online forums devoted to parenting. At least 17.5% of fathers experienced postpartum blues. A high level of education was associated with a higher level of postpartum blues symptoms. Dissatisfaction with the maternity care and significant father involvement during pregnancy and delivery predicted more severe postpartum blues symptoms. Symptoms of postpartum blues were positively correlated with impairment in the father-to-infant bond. This study lends support to the existence of postpartum blues among fathers and highlights its possible consequences on early father–infant relationships.

## 1. Introduction

Perinatal mental health professionals are increasingly aware of the need to detect and to refer women in psychological distress during pregnancy and the postpartum period. But what about men? In France, fatherhood has considerably evolved over the last few decades, as fathers are highly encouraged to become involved in all stages of parenthood [1], from pregnancy to childrearing [2]. These changes have probably induced a greater emotional involvement of fathers in parenthood than in the past [3]. For European first-time fathers, the transition to parenthood requires significant adaptation and good coping skills to face the creation of a new identity, the increase in responsibilities [4], and the required adjustments in the couple’s relationship [5].

Unlike the mother who experiences the child growing inside her, it is often only at childbirth that the baby becomes real to the father [6,7]. Several recent European studies have shown that many fathers report lacking information [4] and may feel unheard, frustrated, and even excluded at the maternity ward [4,7,8], a place still perceived as devoted only to women. According to Boyce et al. [9], up to 18% of Australian and New Zealander fathers experience significant stress in the days following the birth of their child, which could engender transitory psychological difficulties, thus highlighting the importance of giving fathers special attention during this period of upheaval.

Despite growing attention towards fatherhood, studies on fathers in the perinatal period remain scarce [10], while postpartum mental health issues have been widely investigated in women. In this study, we explored, in men, one of the most common postpartum syndromes in women: the postpartum blues.

Postpartum blues (PPB) is defined by Kennerley and Gath [11] as a “brief psychological disturbance in mothers during the first few days after delivery” and includes a mixed mood (alternating depressive mood, often accompanied by tearfulness, and excitement and elation, which can suggest hypomania), emotional lability, slight memory problems, and attention and sleep disorders. Feelings of guilt, anxiety, and irritability are also common features of PPB [11]. PPB symptoms are observed within 10–15 days of birth, are not considered to be a pathological condition, and do not require medication [12].

It is important to understand the differences between PPB and postpartum depression (PPD). PPD is defined as “mental disorder that occurs for the first time within four weeks after delivery” and characterized by “depressed mood and loss of pleasure in daily interested activities” [13]. Unlike PPB, PPD is a mental pathology requiring psychiatric care. However, several studies have found a link between intense or lasting symptoms of PPB and later PPD in mothers [14,15,16]. For instance, 14% of women who experienced a PPB had a PPD 3 months later, and 83% of depressed women have had a PPB [15].

In recent years, PPD in fathers has been increasingly studied. Indeed, the prevalence of postpartum depression in fathers is quite similar to that reported in mothers (between 10 and 15%) and is estimated at 10% [17,18,19]. This prevalence and the links between PPB and later postnatal depression in women underline the need to assess PPB also in fathers.

The PPB has been almost exclusively studied as a syndrome affecting mothers due to its partly presumed hormonal origin [20]. However, the endocrinal hypothesis remains speculative and does not explain why some women have PPB and others do not [21,22,23]. Indeed, on the one hand, PPB has also been described as a marker of affective vulnerability and psychological readjustment in the immediate postpartum period [24,25], thus possibly also affecting fathers [22]. On the other hand, there is some evidence that paternal depression symptoms in the postpartum might be related to hormonal changes, including a decrease in testosterone, cortisol, estrogen, and prolactine levels [26,27,28,29,30]. These physiological changes could engender hypersensitivity towards the newborn in fathers [31]. Thus, there are both psychological and biological reasons that lend support to the existence of PPB in fathers.

A recent meta-analysis found that the prevalence of PPB in mothers varies from 13.7% to 76.0% [32]. Disparities in these rates can be linked to the use of different assessment tools (some are designed to measure postnatal depression) and to the time of assessment. Most studies in women have used the Maternity Blues Questionnaire [11], and there is no validated tool for the assessment of PPB in fathers. To date, the only study on fathers reported a rate of 67% [33], measured by the Maternity Blues Questionnaire. This study found that mothers experienced significantly more blues symptoms than did fathers and that fathers showed PPB symptoms earlier than mothers (peak on day 1 for fathers vs. days 3–4 for mothers). Moreover, Edhborg showed that some clusters identified in PPB are more predictive of PPD than others, and different between men (“primary blues” and “hypersensitivity”) and women (“depression”).

Several psychosocial factors seem to impact the intensity of PPB symptoms in mothers, such as maternal self-esteem and stress related to baby care [34,35], the newborn’s health status and the course of pregnancy [36], social or marital support [37], or economic status [38,39]. All these factors may also apply to fathers’ PPB and deserve to be investigated. Furthermore, research on paternal PPD has identified several risk factors, such as financial instability, unemployment, perceived stress, low education level, history of mental health problems in the father, maternal depression, lack of social support, low confidence in parenting abilities, and dissatisfaction in the relationship [40].

The emotional condition of the father during the postpartum period could have an impact on their initial relationship with their child, particularly regarding father-to-infant bonding. Bonding is a crucial process in the early interactions between parents and their newborn [41], as it is a driving force for parent’s involvement and the development of adapted parental behaviors [42]. Conversely, a failure in the creation of this bond can have deleterious effects on the parent–child relationship and on the child’s emotional development [43,44]. Studies on women have shown that depressive symptoms in the postpartum period (blues or depression) can hinder positive mother-to-infant bonding [45,46,47]. Likewise, several studies on paternal PPD have found a link between depressive and anxiety-related symptoms in the perinatal period and impaired infant bonding [48,49,50].

Despite growing attention towards men’s mental health in the perinatal period, little is known about the prevalence of PPB in fathers, its associated factors, and the potential impact of PPB on parent–child early interactions. Thus, the aims of the present study were (a) to evaluate the prevalence of PPB in fathers, (b) to explore the sociodemographic and perinatal factors that may be associated with its intensity, and (c) to investigate the relationship between the intensity of PPB symptoms and the quality of father-to-infant bonding.

## 2. Materials and Methods

### 2.1. Participants

Participation in this study was proposed to French-speaking fathers from the general population; living in France; and recruited from maternity hospitals, a Child and Maternal Health Centre, and social media in France. Child and Maternal Health Centers are public prevention and follow-up services for parents and children from the time of pregnancy to the child reaching 6 years, and they are present in every French administrative area. Participation took place within 10 days of their infant’s birth. Fathers answered a set of questionnaires, either the paper version or online on Google Forms. The data collection and analysis were carried out in a manner that ensured participant anonymity and confidentiality. Every participant was given detailed information about the research and its objectives and was required to give their consent before completing the survey. In case of any discomfort or distress while filling out the questionnaire, participants were given the option to leave their email address with the principal investigator to follow up and refer them to mental health services if needed. The researchers’ contact information was also provided at the end of the questionnaire. Each participant was assigned a unique code to facilitate data collection and to prevent online participants from responding the survey multiple times. This study was approved by the Research and Ethics Committee of the University of Paris (CER-U-Paris, IRB: 00012021-128).

The participants were recruited from the general population, were aged between 18 and 60 years old, had to be biological fathers of the infant, and has to be fluent in French. Fathers who had experienced infant loss at birth or during the postpartum period, or whose female partners had birth complications requiring hospitalization were excluded from the sample.

### 2.2. Measures

The study gathered the participants’ sociodemographic and perinatal information through an ad hoc questionnaire specifically designed for the postnatal period. The first part was designed to collect general information about the fathers (age, level of education, professional and marital status, and income). The second part assessed obstetrical information (number of children, obstetrical history of the female partner, course of pregnancy and childbirth, breastfeeding or formula feeding, and duration of stay in the hospital), the father’s level of satisfaction with care and guidance received from the maternity staff, as well as the father’s perceived level of involvement during pregnancy and after the childbirth. A father’s involvement score was calculated from the following four variables, assigning 1 point for each positive response (min = 0, max = 4): participation in prenatal follow-ups, taking paternity leave, taking paternity leave for more than 14 days, and participation in the baby’s care after birth.

Postpartum blues symptoms were assessed using the Maternity Blues Questionnaire developed by Kennerley and Gath [11], and translated and adapted into French by Glangeaud-Freudenthal et al. [51]. It is the most widely validated questionnaire used for detecting and measuring postpartum blues symptoms. It consists of 28 items that assess the presence and the intensity of emotions related to postpartum blues symptoms within 10 days following birth. For each item, the participant answers “yes” if the feeling was present and “no” if the feeling was absent that day and then indicates how much change that is from their usual mood on a 5-point scale with anchor points of “much less than usual” to “much more than usual”. In the present study, we used only the “yes” and “no” answers, resulting in scores ranging from 0 to 28, with a high score indicating more intense symptoms of postpartum blues. According to Kennerley and Gath [11], the items cover seven domains or clusters: primary blues (seven items), despondency (three items), depression (five items), hypersensitivity (four items), reservation (three items), decreased self-confidence (three items), and retardation (in the meaning of psychic slowdown; three items). The instructions and some items were adapted for fathers (masculinization of items: “cafardeuse” to “cafardeux”; “pleine de joie” to “plein de joie”; and “désemparée” to “désemparé”).

The Postpartum Bonding Questionnaire (PBQ) [41,52] was used to evaluate father-to-infant bonding. The PBQ is a self-reported questionnaire that gauges the quality of bonding between a parent and their baby and identifies any initial disruptions in this process. The questionnaire includes questions such as “I am afraid of my baby”, “My baby irritates me”, or “I feel close to my baby”. The French adaptation of the PBQ was tested on 1227 mothers and consists of 22 items with sound psychometric characteristics, including robust test–retest reliability [53]. Initially developed for women, this instrument was adapted for fathers (masculinization of items: “je me sens distante de mon bébé” to “je me sens distant de mon bébé”; “je me sens heureuse” to “je me sens heureux”; and “mon bébé me rend anxieuse” to “mon bébé me rend anxieux”). Participants were asked to rate each PBQ item on a five-point Likert scale, which ranges from always (score 5) to never (score 0). Questions pertaining to positive emotions were scored inversely. The French version of the questionnaire yields scores between 0 and 110. A higher score on the PBQ indicates greater obstacles in father-to-infant bonding.

### 2.3. Procedure

In this study, fathers were recruited either directly in two maternity hospitals and one Child and Maternal Health Centre in the Paris area, or on social media in France. In the maternity hospital, fathers were approached in the postnatal ward during visiting hours within the first 10 days following childbirth. They were given an envelope so that they could return questionnaires anonymously to the maternity staff. In the Child and Maternal Health Centre, the questionnaires were filled out by the fathers also within 10 days after the birth, during the infant’s weighing routine appointments. The questionnaires were then returned to the staff in an envelope. For the online survey, announcements were posted on maternity hospitals’ social media pages and French parenting forums (“Baby boom”, “Supers parents”, “Bientôt maman”, and “Bons plans et astuces de parents”), and interested fathers were directed to the online survey on Google Forms. Each participant was asked to read the information form and to then complete the Sociodemographic Questionnaire, the Maternity Blues Questionnaire, and the Postpartum Bonding Questionnaire. The average completion time was 20 min. There were no missing data, and all questionnaires were fully completed. Recruitment took place over a 4-month period from mid-December 2018 to mid-April 2019.

### 2.4. Data Analyses

Descriptive and inferential analyses were performed using Statistica 13.3. Given the adequate analyses of normality with Shapiro–Wilk tests, the number of participants (*n* = 303), and the homogeneity of the variances, parametric tests could be carried out. Cronbach’s alphas have been calculated to check the internal consistency of the MBQ (α = 0.824) and the PBQ (α = 0.856) in this sample. With regard to the prevalence of PPB, the absence of an official cut-off score required comparison with the data of other studies, as proposed by Glangeaud-Freudenthal et al. [51]. The study by Edhborg [33] is, to date, the only one to have proposed the Maternity Blues Questionnaire to a large cohort of fathers (*n* = 133). In their study, a score of 6 was retained as the threshold for PPB, based on a mean percentage score over 5 days during the first week postpartum, following the Kennerley and Gath [11] method. The parents were identified as having postpartum blues if the mean percentage scores were above the peak score, i.e., the highest mean percentage score for the whole sample of fathers on any of the 5 days. Studies on women [15,36,54] have established a cut-off score of 12 for moderate blues and 20 for severe blues.

To investigate the impact of sociodemographic and perinatal factors on the intensity of PPB symptoms, t-tests for independent samples, one-way ANOVAs, and multiple regression analyses were performed. Finally, Pearson correlations were performed to investigate the relationship between PPB and bonding scores in our sample.

## 3. Results

### 3.1. Sociodemographic and Perinatal Sample Characteristics

A total of 303 fathers participated in this study; 8.6% of them completed the paper version and 91.4% completed the online version (Table 1). Fathers’ mean age was 32 years (min = 20, max = 52; SD = 5.19). Most fathers (87%) were born in France, 62% had an undergraduate degree at least, and 94% were professionally active (44% employees, 27% executives, 7% independent professionals, 7% factory employees, 4% craftsmen, 3% civil servants, and 1% farmers). All fathers were cisgender and heterosexual, and almost all (98%) were in a relationship at the time of the study, with an average relationship duration of 7 years (36% married, 32% civil union, and 30% cohabiting).

Regarding obstetrical and neonatal data, 67.3% of participants were first-time fathers, 86% of couples planned the pregnancy, and 8% underwent assisted reproduction techniques. Concerning the present pregnancy, 25% of father reported complications during the pregnancy for their partner (threatened preterm delivery, gestational diabetes, and hypertension) and 25% reported complications at childbirth. Most women (78%) had vaginal childbirth (6% forceps- or vacuum-assisted) and 22% had a C-section (7% emergency C-section). Most infants were born healthy, but 15% required some neonatal care at birth and 6% were hospitalized in a neonatology unit. Breastfeeding was chosen by 75% of parents.

As far as fathers’ involvement is concerned, the participants attended on average two types of prenatal appointments: 98% attended ultrasound scans, 62% attended childbirth preparation sessions, and 59% attended a prenatal interview with a midwife or a gynecologist. Most fathers (77%) took a paternity leave, of an average of 17 days. A large majority of fathers (85%) declared that they participated in the baby’s daily care. Finally, 79% were satisfied with the support and advice provided at the maternity hospital, 17% were moderately satisfied, and 4% were dissatisfied.

### 3.2. Prevalence of Postpartum Blues among Fathers

A mean score of 7.26 on the Maternity Blues Questionnaire was found in our sample (min = 0; max = 22) (Table 2). As no cut-off score is determined for the French fathers’ population, we chose to rely on two criteria to describe these results. If we consider the threshold score of 12 used in several studies on mothers [15,36,54], 17.49% of fathers showed PPB symptoms (*n* = 53), including 1.32% (4 participants) with “severe” blues (score greater than or equal to 20). If we use the cut-off score of 6 calculated by Edhborg [33] with Swedish fathers, 58.75% (*n* = 178) of the fathers in our sample can be classified as having PPB.

Regarding the seven domains that constitute PPB according to Kennerley and Gath [11], it appears that fathers in our sample experience more symptoms of retardation and primary blues, as well as hypersensitivity and decreased self-confidence, but in a smaller proportion. The reservation, depression, and despondency domains seem less relevant in characterizing PPB in fathers of our sample.

### 3.3. Factors Associated with Postpartum Blues in Fathers

Among the sociodemographic factors (Table 1), only the fathers’ level of education was associated with the PPB score. Participants with a higher education level had significantly higher levels of PPB symptoms (*p* = 0.046). Contrariwise, the parity and none of the perinatal factors were associated with the intensity of PPB symptoms. However, fathers’ involvement during pregnancy and at birth, as well as their satisfaction with the maternity hospital, were associated with PPB symptoms.

A multiple regression analysis including the level of involvement and satisfaction as predictors of the severity of PPB symptoms showed a significant global model (*p* = 0.012) (Table 3). A high level of involvement during the pregnancy and with the baby, and a low level of satisfaction with the support and advice provided by the maternity staff significantly predicted more intense PPB symptoms. However, this model predicted only a small part of the variance in the PPB score, highlighting the need to explore other factors that may be associated with levels of PPB.

### 3.4. Quality of Bonding and Links with the Intensity of PPB Symptoms

Fathers in our sample had a mean score of 13.6 (min = 0, max = 56) on the PBQ questionnaire. More than half of the fathers (51.5%) had a score higher than the cut-off score of 10 found in the French validation study on mothers [53], meaning that they were experiencing bonding difficulties.

Positive correlations were found between father’s global scores of PPB symptoms and bonding disturbances (r = 0.376; *p* ˂ 0.001). As shown in Table 4, all domains but one (Reservation and Retardation) of PPB were also positively correlated to the PBQ score (*p* ˂ 0.005), thus corroborating the links between PPB symptoms and difficulties in the early relationship with the child. More precisely, the strongest correlations were found between the bonding score and the depression and decreased self-confidence domains of the Maternity Blues Questionnaire (r = 0.316; *p* ˂ 0.001 and r = 0.264; *p* ˂ 0.001, respectively).

## 4. Discussion

This study aimed to assess the prevalence of PPB in a sample of French-speaking fathers living in France, as well as the relationship between its intensity and sociodemographic factors, perinatal factors, and bonding quality during the first 10 days postpartum. This study contributes to filling the gap in research on fatherhood in the peri-partum period [10] and expands existing knowledge on the links between PPB and bonding in new parents [46,55,56].

### 4.1. Prevalence of PPB in Fathers

Our first aim was to investigate the prevalence of PPB in a sample of French-speaking fathers. The results underline the existence of PPB in at least 17.5% of fathers if the same cut-off score of 12 is chosen as for women, which questions its female specificity. Furthermore, if Edhborg’s [33] cut-off score is used, established in a sample of fathers and as recommended by Glangeaud-Freudenthal et al. [51], a rate of 58% of the fathers would be likely to show PPB. This result indicates that more than one father out of two would present typical signs of PPB, a rate that is close to that generally found in mothers. Moreover, the results obtained in the sub-domains of the Maternity Blues Questionnaire are in line with those observed in mothers by Kennerley and Gath [11] and those found by Edhborg [33] in his comparative study of fathers and mothers. The most intense symptoms of PPB are indeed similar in men and women, which supports the relevance of investigating PPB symptoms in fathers too.

### 4.2. Associated Factors

Our second aim was to identify, among a large array of sociodemographic and perinatal factors, those that may be associated with the intensity of PPB in fathers. Similarly to several studies conducted amongst mothers [15,36,39], most sociodemographic characteristics were not related to the intensity of PPB symptoms. Surprisingly, only the fathers’ level of education being higher was associated with higher PPB symptoms. A recent study by Gerli et al. [57] on mothers have found the opposite: women with lower levels of education had on average higher levels of PPB. Further studies remain to be conducted on sociodemographic factors and on similarities and differences between men and women related to these factors and PPB symptoms.

With regard to obstetrical factors, neither parity nor obstetrical complications seemed to be related to the intensity of PPB symptoms. Similar results have been found in studies on women’s samples [14,15,35]. However, the fathers’ level of involvement during pregnancy and birth, and level of satisfaction with the support and advice provided by the maternity staff were both significantly associated with the occurrence of PPB. The more involved fathers were, the higher the PPB symptoms, and the more satisfied they were, the less severe the PPB symptoms. The link between fathers’ involvement and PPB could support the idea that blues symptoms have an adaptative function in a period of emotional upheaval [20]. It can be hypothesized that fathers who are more physically involved are also more emotionally affected. A longitudinal study exploring the link between PPB symptoms and fathers’ involvement could help to enlighten their evolution in time. Concerning the level of satisfaction, a lack of information and support regarding the childbirth and childcare and a feeling of not being able to express emotions and needs could impact new fathers’ wellbeing and engender psychological distress [4]. The lack of support may also lead to a feeling of exclusion among fathers [7] and affect the men’s self-esteem as fathers [58]. Taken together, these feelings may increase fathers’ distress and contribute to PPB symptoms. Studies on mothers have highlighted the impact of a high level of anxiety and low self-esteem as a mother on the intensity of PPB symptoms [34,35]. Further studies could therefore look into the links between fathers’ level of anxiety, paternal self-esteem, and the intensity of PPB symptoms.

### 4.3. Impact on Father-to-Infant Bond

Our third aim was to investigate the possible links between the intensity of PPB symptoms and the quality of father-to-infant bonding in the first days after the childbirth. Significant positive correlations between the global scores of PPB and bonding were found. Thus, in our sample, the more intense the PPB symptoms, the lower the quality of the father–infant bond. The depression and decreased self-confidence domains of the Maternity Blues Questionnaire were the most related to the quality of bonding. These findings are consistent with the existing literature on mothers [45,46,47] and highlight the negative impact of PPB symptoms on the quality of father-to-infant bonding.

### 4.4. Limitations

Several limitations deserve to be acknowledged in this study. First of all, the lack of validated tools for the assessment of PPB and bonding in fathers has led us to adapt questionnaires validated for women, and this might have biased our data. Wording of the items had to be modified in order to adapt them to the fathers’ experience; thus, one could question its possible impact on PPB and bonding scores. Indeed, there is an urgent need to validate or to develop accurate and specific mental health assessment and diagnostic tools for fathers. Secondly, although we sought to recruit fathers from all segments of the population, our final sample is composed of cisgender fathers with a high level of education, most of whom work, are in a heterosexual relationship, and experienced non-complicated childbirth. In addition, this study was conducted on French-speaking fathers living in France. Thus, our findings are not generalizable to other groups from diverse sociodemographic and cultural backgrounds. Moreover, due to the recruitment modalities and the study design, we were unable to ask parents to complete the Maternity Blues Questionnaire on each of the first 10 days, preventing us from establishing a mean score over this period and detecting a peak of PPB symptoms. For the measurement of PPB symptoms, we also compared our results with existing thresholds for women in the literature. In addition to using validated tools, interviewing fathers in the immediate postpartum period is crucial to determine specific symptoms and a cut-off score of PPB for fathers and to bring to light father-specific factors that have not been considered in mothers. Moreover, the use of a cut-off enables a categorical approach to PPB but remains questionable. A few studies using the MBQ chose not to, considering the interest of a more descriptive approach rather than a categorical one, especially with a clinical objective in mind. It would also be relevant to evaluate the fathers’ level of satisfaction with the support provided by the maternity staff more precisely in order to better target the origin of their dissatisfaction that contributed to PPB symptoms, as found in the present study.

More generally, some questions remain regarding the differences between mothers’ and fathers’ peri-partum experiences. These differences might concern not only the intensity of blues symptoms but also the types of emotions expressed. For instance, fathers could show more anxious than depressive symptoms compared with mothers, as observed by Matthey et al. [59]. This points out the relevance of assessing postnatal anxiety in fathers and its links with PPB in future studies. If PPB manifestations are different in fathers, one could then wonder about the relevance of bringing together two different clinical entities under the same label. However, keeping the term postpartum blues has two advantages. Firstly, it regroups these clinical manifestations which develop at approximately the same time, as observed by Edhborg [33], in the very specific context of the immediate postpartum period. Second, it has the advantage of being relatively well-known; thus, it may be a better communication vector when it comes to prevention among the general population and health professionals.

## 5. Conclusions

This study confirms that more attention should be given to fathers in the postnatal period. Our results bring to light the psychological fragility of more than half of the fathers in our sample in the 10 days following childbirth, and its potential negative impact on father-to-infant bond. However, in France and in many other countries, very few maternity hospitals are prepared to welcome and take care of future and new fathers. In some maternity hospitals, arrangements have been made to integrate fathers more fully into the perinatal follow-ups and to provide them with better support. In France, some maternity services have recently developed the “Daddy-Friendly” chart, which includes specific psychological support and discussion groups for future and new fathers. These initiatives seem promising and valuable for preventing psychological difficulties in the peri-partum period, such as those demonstrated for the father-to-infant bond in case of prematurity [60,61].

Finally, it is important to bear in mind that PPB is not a pathological condition. Thus, its existence in fathers seems rather natural and highlights the importance of providing them with time and attention in the days following the birth of their child. Informing the maternity staff and primary care professionals about the existence of PPB in fathers and the ways to provide support and reassurance is crucial to offer fathers and their infant the best start possible in the postpartum period. While early support allows mothers to surmount PPB naturally [20], fathers still rarely benefit from such support, increasing the risk of developing more severe PPB symptoms that can lead to later paternal postpartum depression.

## Figures and Tables

**Table 1:** Links between sociodemographic and perinatal factors, and the intensity of postpartum blues symptoms.

Variables	Participants *n* = 303 (%)	Mean Score at MBQ (SD)	*t-Test or F*	*p Value*
**Age**				
<32 years	151 (49.83)	7.20 (4.61)	−0.218	0.827
≥32 years	152 (50.12)	7.32 (4.72)		
**Place of birth**				
Europe	281 (92.74)	7.17 (4.61)	−1.157	0.248
Other	22 (7.26)	8.36 (5.20)		
**Level of education**				
Undergraduate or postgraduate	188 (62.05)	7.68 (4.83)	2.007	0.046 *
A-level or less	115 (37.95)	6.57 (4.30)		
**Professional status**				
Active	284 (97.73)	7.05 (4.61)	−3.047	0.003 *
Inactive	19 (2.27)	10.37 (4.32)		
**Duration of couple relationship**				
≤6 years	169 (55.78)	7.27 (4.67)	0.037	0.97
>6 years	134 (44.22)	7.25 (4.67)		
**Parity**				
Primiparous	204 (67.33)	7.15 (4.55)	−0.565	
Multiparous	99 (32.73)	7.47 (4.90)		0.573
**Partner’s history of obstetrical complications**				
Yes	86 (28.38)	7.19 (4.83)	0.168	0.867
No	217 (71.62)	7.29 (4.60)		
**Assisted reproductive techniques**				
Yes	25 (8.25)	7.76 (4.48)	−0.562	0.574
No	278 (91.75)	7.21 (4.68)		
**Planned pregnancy**				
Yes	252 (83.17)	7.25 (4.63)	−0.124	0.901
No	44 (16.83)	7.34 (4.90)		
**Childbirth**				
Vaginal	218 (71.95)	7.21 (4.80)	1.230	0.299
Vaginal forceps-vacuum assisted	19 (6.27)	7.47 (3.42)		
Planned C-section	45 (14.85)	6.62 (4.47)		
Emergency C-section	21 (6.93)	8.95 (4.40)		
**Obstetrical complications**				
Yes	119 (39.27)	7.74 (4.48)	−1.451	0.148
No	184 (60.73)	6.95 (4.76)		
**Breastfeeding**				
Yes	226 (74.59)	7.32 (4.72)	−0.39	0.696
No	77 (25.41)	7.08 (4.50)		
**Participation in baby’s care at birth**				
Yes	45 (14.85)	8.49 (4.31)	−1.93	**0.055**
No	258 (85.15)	7.04 (4.69)		
**Stay at hospital**				
≤5 days	257 (84.72)	7.08 (4.71)	−1.555	0.121
>5 days	46 (15.18)	8.24 (4.27)		
**Participation in prenatal follow-ups**				
≤2 types of follow-ups	184 (60.73)	7.33 (4.92)	0.344	0.731
>2 types of follow-ups	119 (39.27)	7.14 (4.24)		
**Paternity leave**				
Yes	234 (77.23)	7.41 (4.73)		
No	69 (22.77)	6.75 (4.40)	−1.022	0.308
**Planned length of paternity leave**				
≤14 days	229 (75.58)	7.04 (4.57)	−1.436	0.152
>14 days	74 (24.42)	7.93 (4.91)		
**Participation in baby care**				
≤2 types of care	262 (86.47)	7.19 (4.64)	−0.628	0.53
>2 types of care	41 (13.53)	7.68 (4.84)		
**Satisfaction with maternity staff**				
Satisfied	238 (78.55)	6.92 (4.55)	F = 3.399	0.035 *
Moderately satisfied	52 (17.16)	8.77 (4.98)		
Dissatisfied	13 (4.29)	7.31 (4.46)		

Note: MBQ: Maternity Blues Questionnaire; SD: Standard Deviation; * *p* < 0.05.

**Table 2:** Descriptive sample characteristics in the Maternity Blues Questionnaire.

	Participants (*n* = 303)
**Total score**	
Mean (SD)	7.26 (4.66)
Min	0
Max	22
**Postpartum blues relative to cut-off scores (%)**	
≥12	53 (17.49)
>12	250 (82.51)
≥6	178 (58.75)
>6	125 (41.25)
**Sub-scores M (SD)**	
Retardation	0.39 (0.32)
Primary blues	0.37 (0.22)
Hypersensitivity	0.28 (0.27)
Decreased self-confidence	0.28 (0.33)
Reservation	0.17 (0.24)
Depression	0.14 (0.20)
Despondency	0.11 (0.15)

Note: M = Mean; SD: Standard Deviation.

**Table 3:** Fathers’ level of involvement and satisfaction with maternity staff as predictors of postpartum blues intensity.

Variables	β	*t (299)*	*p*
Level of involvement	0.11	1.78	0.077
Level of satisfaction	−0.147	−2.57	**0.011**

Global model: R^2^ = 0.022; ***p* = 0.012**.

**Table 4:** Correlation matrix between postpartum blues sub-scores and bonding score in PBQ.

	Primary Blues	Reservation	Hypersensitivity	Depression	Despondency	Retardation	Decreased Self-Confidence
**PBQ Score**	0.161 *	0.168 *	0.198 **	0.316 **	0.220 **	0.169 *	0.264 **
Primary blues	—	0.339 **	0.602 **	0.472 **	0.246 **	0.190 **	0.388 **
Reservation		—	0.427 **	0.466 **	0.168 *	0.11	0.250 **
Hypersensitivity			—	0.483 **	0.223 **	0.190 *	0.410 **
Depression				—	0.150 *	0.191 **	0.256 **
Despondency					—	0.243 **	0.363 **
Retardation						—	0.392 **
Decreased self-confidence							—

Note. ** *p* < 0.001; * *p* < 0.005; PBQ = Postpartum Bonding Questionnaire.

## Data Availability

Data from this study are not available due to restrictions imposed by the Ethics Committee.
